# Solutions to Neglected Tropical Diseases Require Vibrant Local Scientific Communities

**DOI:** 10.1371/journal.pntd.0000662

**Published:** 2010-03-30

**Authors:** Serap Aksoy

**Affiliations:** Yale School of Public Health, Epidemiology and Public Health, New Haven, Connecticut, United States of America

To advance worldwide health, it is necessary to build and strengthen scientific and research capacity. In turn, for science to become maximally influential and globally productive, vibrant local science communities are essential, particularly in developing countries. Local communities worldwide are especially necessary for effective control of neglected tropical diseases, which disproportionately affect the populations living in low- and middle-income countries [1]. In the past decade, scientific research capacity of resource-limited countries has increased substantially through growing philanthropy and public–private partnerships as well as a revised strategic focus of funding agencies. The presence of a visible and informed local research community is indispensable in the mutual trust-building and shared decision-making that are needed as discoveries make their way from bench to practice. An effective means of advancing such capacity is for local scientific communities to gain visibility and credibility through publication of their research findings. Unfortunately, most scientists in low- and middle-income countries continue to remain at the periphery of this critical communication highway.

Much has been written about scientific capacity, yet a universal definition has remained elusive. Transfer of technology, tools, and knowledge are clearly important building blocks for advancing research capacity, as is the establishment of vibrant local scientific communities. Although scientific research is pivotal in the process of translating evidence-based interventions into better health outcomes for the population, successful implementation of such health interventions in disease-endemic setting has varied. In fact, one of the significant challenges to improving health in the general population, particularly in low- and middle-income countries, continues to be sustainable adoption and implementation of evidence-based interventions. An upcoming Viewpoint article by Lang and colleagues in *PLoS Neglected Tropical Diseases* discusses the urgent need for building clinical research capacity in disease-endemic countries to lead clinical trials for new treatments and vaccines [2]. Successful implementation of health interventions requires not only highly trained professionals with great skills and expertise, but also close communication between the research community and health care professionals, health care organizations, consumers, and policy-makers. Such essential communication includes dissemination of information, particularly of research findings relevant to local problems and solutions.

For maximal dissemination and global recognition of local scientific communities, research papers need to be published in journals that are not only peer-reviewed but also included in global indexing databases, such as in those of the Institute for Scientific Information (ISI). In addition, participation of scientists and scholars of these communities in the global scientific network as editors and peer-reviewers provides them opportunities to exchange ideas, establish global collaborations with their peers, and exchange students.

One of our main goals at *PLoS Neglected Tropical Diseases* is to promote and profile the efforts of scientists in disease-endemic countries and to help build science and health capacity in these countries. We hope to achieve our mission by giving scientists open access to essential information and by providing a venue for publishing their research. Therefore, we are keen to publish research articles contributed by authors working at institutions in low- and middle-income countries where these diseases have the greatest impact. To integrate scientists and scholars from disease-endemic country into all aspects of the journal's activities, we also promote a strong representation of associate editors from low- and middle-income countries. Associate editors are responsible for overseeing the peer-review process, and they work closely with our deputy editors and journal management staff on all aspects of the review process and decision outcome. In fact, 37% of our associate editors are scientists and/or scholars from disease-endemic countries.

**Figure 1:**
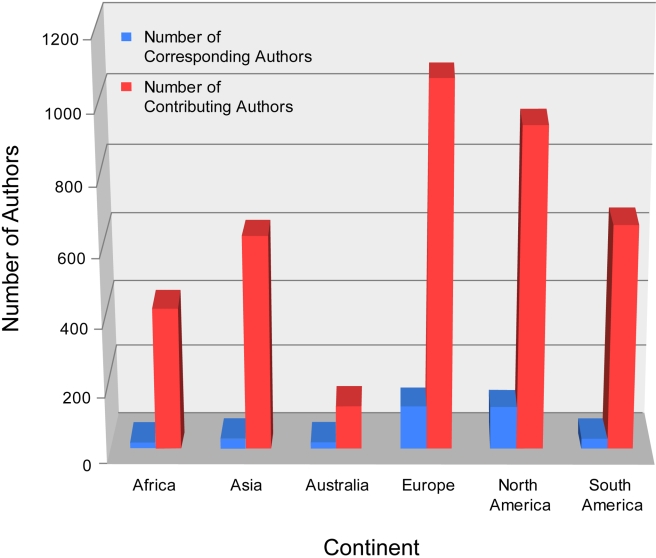
Continent-based analysis of authorship in *PLoS Neglected Tropical Diseases* articles through December 2009.

*PLoS Neglected Tropical Diseases* recently analyzed the papers published in the journal through December 2009. In particular, we compared the statistics on the number of contributing authors versus the number of corresponding authors associated with these papers based on their continental distribution. Although corresponding authors are essential for generating the data, the contributing author is often the lead scientist for the project development, management, and manuscript writing processes. Our data, summarized in Figure 1, show that among corresponding authors of the papers we have published to date, about 80% reside in an industrialized nation in North America, Europe, or Australia. Only 5% of the corresponding authors are from an African institution, with the majority being from South Africa.

Although among contributing authors, 40% reside in disease-endemic countries (in Africa, Asia, and South America), only 10% are from an African institution. Given the disproportionate impact of neglected tropical diseases in Africa and the excellent work that goes on in many institutions from that continent, we hope to encourage greater participation from scientists that reside where public-health–related improvements are most needed.

An important deterrent to greater submissions from disease-endemic countries is the scarcity of information available to young researchers on the publication enterprise, from the crafting of a manuscript to the submission process. Conversations I have had with my collaborators in Africa have made me realize that many students and junior scientists are unaware of the various editorial and technical support systems available to young authors these days. To bring more transparency to the publication process, we at *PLoS Neglected Tropical Diseases* are eager to reach out to scientists all over the world about this process and convey the importance of publishing their data to promote the global scientific progress.

On our Web site (http://www.plosntds.org/static/downloads.action), you will find a copy of a recent presentation on a Manuscript Writing Workshop I recently held. This presentation discusses how to avoid some of the most common mistakes that result in rejection of manuscripts. It also gives some tips on effective writing skills and the scientific format we follow for research articles. In addition, it provides information—especially aimed at junior scientists—on the various editorial resources available to help improve manuscript writing skills. Our take-home message is that if you are addressing an important question, if you have used the correct methodology to analyze your data, and if you are succinctly and clearly conveying your findings, you can be confident that your manuscript will be seriously considered for publication. We look forward to reviewing your work!
